# Accommodation for Maternity Cases

**Published:** 1920-03-20

**Authors:** 


### The Hospital

The Workers' Newspaper of Administrative Medicine and Institutional Life, Administration, National Insurance and Health.

No. 1763, Vol. LXVII. SATURDAY, MARCH 20, 1920. PRICE TWOPENCE.

### ACCOMMODATION FOR MATERNITY CASES.

A short time ago we published a note upon the need for improved obstetric teaching and a few methods by which it might be attained. Although this is a subject which has already received a great deal of consideration in recent times, we offer no apology for again bringing it to notice, in that its importance is so great that in no circumstances must the problems it involves be again shelved and forgotten.

Quite apart from the direct teaching of obstetrics and gynæcology to medical students and graduates in London, the subject, fully discussed at a meeting of the particular section of the Royal Society of Medicine held on December 4, involves the necessity for providing more beds for lying-in women throughout the whole country and a far better organisation for their medical attention. The discussion mentioned dealt only with maternity accommodation in London, but we may take the conditions in London as being a typical example of the state of affairs in most of the large British cities; suffice it to say that the conclusions of the section make it clear beyond all doubt that hospital accommodation for lying-in cases is practically everywhere totally and absolutely inadequate.

In his now famous memorandum dealing with the practice of preventive medicine, Sir George Newman, too, approached the subject from an aspect other than the educational, and made it perfectly clear that the great centres of population are in need of two distinct organised maternity provisions. They must have maternity *hospitals* or hospital accommodation for abnormal or doubtful cases, and maternity *home* accommodation for the enormous number of perfectly normal cases among the poor which cannot, however, be normally or even safely cared for in the overcrowded, insanitary and utterly unsuitable tenements which still abound in this country. In relation to their actual needs the rural districts are not one fraction better, and it is worth recalling the figures which Sir George suggested as the minimum provision we should demand. One maternity bed to 2,000 of the population, the whole being attached to already established institutions if possible, and providing a small general ward and an isolation or observation section. Also, once this development is actually set going and units with beds, local hospital connections and adequate nursing staffs are provided, not only will the very poor have received one of the greatest blessings which can come their way, but also there will become possible further extensions towards truly modernised and efficient pay-institutions of moderate charges, which are now a great, and must in future be a yet greater, requirement for the poorer middle classes. Some may say we have rubbed along fairly well in the past, that voluntary aid and charities have done much and willing workers have to some extent kept pace with the times. But nowadays, with an again increasing population, an improved natural efficiency as a nation absolutely necessary, and the memory of terrible revelations that the war period brought to our notice concerning the true state of the nation's physique and general fitness, the problem becomes one of such vital importance that rapid, centralised Government activity in the matter is absolutely essential.

The whole questions both of teaching and of accommodation are so intimately interwoven that it is not only impossible to consider them separately, but also impossible that they should be separately dealt with in practice. Actually the requirements in available beds for instruction of students and graduates may be definitely known, but it is probable that if this number be compared with that needed by the public, then the latter or the true preventive medicine aspect of the question makes by far the greater demand. When there is sufficient accommodation, in their time of need, for the mothers and potential mothers of the country, then there will be no lack of teaching possibilities in even the smaller towns and cities.

For this reason, we would prefer that the first developments should be made along lines guided mainly by considerations such as Sir George Newman's report formulates. Suggestions already made by the Ministry of Health have, as is well known, received a mixed reception owing to criticisms coming from quarters not primarily interested in the public health side of the matter. Yet all are agreed that more and yet more accommodation for maternity and allied cases we must have, and to us differences appear to arise mainly in the exact method of bringing them into being, their disposal, and the origin and distribution of the necessary monies. When the time for final decision arrives, and it is to be hoped that this is now not very far distant, we feel that the plan from which the country as a whole will derive the greatest benefit is that which aims at solving in the first place the problems arising directly with the masses themselves. The medical educational aspect should thereafter solve itself automatically, for then material galore for observation will be there, and mere organisation of academic training will readily bring the medical world, young and old, into contact with it when necessary. Let us start right off by providing mothers and nursing mothers with the care they need; there is enough trained medical and nursing talent fully to equip the first institutions organised, and the future medical generations will rapidly come to keep pace with the movements of the times. On a later page (p. 583) we review the latest constructional suggestions.

